# Effects of variability in daily light integrals on the photophysiology of the corals *Pachyseris speciosa* and *Acropora millepora*

**DOI:** 10.1371/journal.pone.0203882

**Published:** 2018-09-21

**Authors:** Stephanie DiPerna, Mia Hoogenboom, Sam Noonan, Katharina Fabricius

**Affiliations:** 1 College of Science and Engineering, James Cook University, Townsville, Queensland, Australia; 2 ARC Centre of Excellence for Coral Reef Studies, James Cook University, Townsville, Queensland, Australia; 3 Australian Institute of Marine Science, Townsville, Queensland, Australia; National Research Council of Italy, ITALY

## Abstract

Phototrophic sessile organisms, such as reef corals, adjust their photosynthetic apparatus to optimize the balance of light capture versus protection in response to variable light availability (photoacclimation). In shallow marine environments, daily light integrals (DLI) can vary several-fold in response to water clarity and clouds. This laboratory study investigated the responses of two coral species to fluctuations in DLI. Corals were exposed to four contrasting DLI treatments: ‘high-light’ (potentially photoinhibiting conditions, 32 mol photons m^-2^ d^-1^), ‘low-light’ (potentially light-limiting conditions, 6 mol photons m^-2^ d^-1^), and two ‘variable light’ treatments that alternated between high and low conditions every 5 days. In the variable treatments, the shade-tolerant coral *Pachyseris speciosa* displayed cycles of rapid declines in maximum quantum yield during high-light and subsequent recoveries during low-light, showing photoacclimation at a time scale of 3–5 days. In contrast, the shallow-water coral *Acropora millepora* showed slow (>20 days) photoacclimation, and minimal changes in photosynthetic yields despite contrasting light exposure. However, growth (change in buoyant weight) in *A*. *millepora* was significantly slower under variable light, and even more so under low-light conditions, compared with high-light conditions. The responses of yields in *P*. *speciosa* match their preference for low-light environments, but suggest a vulnerability to even short periods of high-light exposure. In contrast, *A*. *millepora* had better tolerance of high-light conditions, however its slow photoacclimatory responses limit its growth under low and variable conditions. The study shows contrasting photoacclimatory responses in variable light environments, which is important to identify and understand as many coastal and midshelf reefs are becoming increasingly more turbid, and may experience higher variability in light availability.

## Introduction

Phenotypic plasticity can increase the fitness of organisms by enabling them to cope with variable environmental conditions [1, 2]. Plasticity can be based on phenotypic expression during development (developmental acclimation) or from reversible and dynamic changes in response to changes in environmental conditions [1]. In shallow water environments, light reaching the seafloor (benthic irradiance) is highly variable. Light fluctuates within seconds through wave lensing, but also throughout the day due to sun angle and intermittent cloud cover [3]. Variable clouds and turbidity can alter the cumulative amount of light a benthic organism receives (daily light integrals; DLI) up to five-fold from one day to the next [4], on top of seasonal changes due to varying day length and sun angle [5]. As such, benthic marine organisms grow in constantly changing light environments. Light intensity can range from suboptimal for maximal photosynthesis rate (light-limiting conditions) up to levels that cause damage to the photosynthetic apparatus (photoinhibitory conditions) [6, 7]. Plasticity that optimizes photosynthesis under different light intensities is known as photoacclimation, enhancing the fitness of phototrophic organisms [1, 8] and affecting ecosystem functioning and biodiversity [9].

For sessile photosynthetic organisms in marine environments, such as corals, seagrasses and algae, the ability to photoacclimate is critical to their survival and growth. Reef-building corals use a combination of morphological and physiological strategies, manifest by both coral host and symbiotic dinoflagellates (together termed the coral holobiont), to photoacclimate in variable light environments. Over their lifetime, coral colonies can change their morphology to optimize light exposure [10]. At time scales of days to weeks, the coral host also physiologically adjusts concentrations of photoprotective and antioxidant compounds within their tissue [11, 12]. However, much of corals’ capacity for short-term photoacclimation is driven by adjustment of various components of the photosynthetic apparatus in the symbiotic dinoflagellates [13]. For instance, the coral holobiont can photoacclimate via changing the size and number of photosynthetic units (PSU) in the symbionts to optimize absorption of light energy [14, 15]. This can include increasing the concentration of photosynthetic pigments (such as chlorophyll *a*, *c*_*2*_ and peridinin) to increase light harvesting ability, and increasing photoprotective pigmentation (such as xanthophylls and β-carotene) to dissipate excess photon energy and/or act as antioxidants to combat destructive reactive oxygen species formed during high-light exposure [13, 16, 17]. Such changes ensure sufficient light energy is harvested under light-limiting conditions while reducing damage under photoinhibiting conditions, thereby maximizing photosynthetic energy gains for growth and reproduction.

Different coral species use different strategies for photoacclimation (physiological versus morphological changes), and strategies depend on the direction of change in light. For instance, Browne et al [18] showed that *Pachyseris speciosa* and *Merulina ampliata* responded rapidly to decreased light availability by increasing the photosynthetic potential, or maximum quantum yield of photosystem II (F_v_/F_m_), whereas *Platygyra sinensis* did not respond. Other studies also indicate a general increase in F_v_/F_m_, after a decrease in light intensity in the environment [6, 14, 19]. Similarly, Anthony and Hoegh-Guldberg [7] compared *Turbinaria mesenterina* photosynthesis to irradiance curves (P-I curve) constructed from oxygen-respirometry techniques to assess the short-term change in the irradiance level at which photosynthesis becomes saturated (saturating irradiance, denoted as I_k_) when transitioning from high-light to low-light and vice versa. Irradiance levels that are higher or lower than I_k_ can reduce the organism’s efficiency to capture and utilize incoming light energy, so being able to quickly adjust this parameter demonstrates photosynthetic flexibility to changing environments [20].

To date, many studies examining photoacclimation in reef corals have focused on photoacclimatory responses after a single transition event between two constant light environments (e.g. [7, 21, 22], or else they compared deep versus shallow water corals [23, 24]. While these studies have greatly expanded our understanding of photoacclimation in reef corals, they provide limited insight into photoacclimation under the fluctuating light conditions that occur in nature. Studies on other photosynthetic organisms, such as phytoplankton and higher plants, reveal they photoacclimate to the DLI, regardless of how variable the light is during the day period [25, 26], or that they photoacclimate to average light conditions when light fluctuates faster than acclimation rates [27, 28]. In addition, other studies suggest photoacclimation is asymmetrical between acclimation to high versus low-light, as high-light intensities can rapidly damage pigments and proteins involved in photosynthesis, and their repair is slow [24, 29, 30], which slows rates of acclimation.

No coral studies thus far have directly compared coral photoacclimation and performance under fluctuating and fixed light environments. For plants, however, a study by Mischra et al [31] found *Arabidopsis* plants grown indoors with relatively consistent DLI had a reduced ability to cope with high-light exposure compared to plants grown under natural sunlight. For corals, indirect evidence of changes in photoacclimation capacity under fixed light conditions comes from studies showing that photosynthetic potential (F_v_/F_m_) is often lower in the field or under outdoor laboratory experiments [32] compared to fixed lighting laboratory studies [22, 33] at similar DLI. The aim of this study was to understand photoacclimatory patterns of two reef coral species, the plating *Pachyseris speciosa* and branching *Acropora millepora*, to diurnal light profiles that either did not change from day to day (referred hereto as constant conditions), or that varied between days (variable conditions). The latter mimicked changes in benthic irradiance for several days resulting from pulses of turbidity or overcast periods. We aimed to investigate the general mechanisms and capacity of corals with contrasting morphologies to deal with this type of variable light, as seen in many shallow reef environments. Specifically, the study investigated whether corals exposed to variable conditions (i) acclimate to the average, lowest or highest DLI they experience, or constantly and rapidly adjust to the changing light regimes. The study also addressed (ii) how rapidly the corals adjust their photosynthetic processes to their new light environment when transferring from high-light to low-light and vice versa, (iii) whether levels of light stress (either photoinhibition or light-limitation) decrease after repeated exposure under variable conditions, and (iv) the implications of variable light on the corals’ net oxygen production and growth rates.

## Methods

### Experimental setting

Eight partial colonies each of *Pachyseris speciosa* (from 5–8 m depth) and *Acropora millepora* (from 3–5 m depth) were collected from Davies Reef, central Great Barrier Reef, Australia in July 2016, and taken to outdoor flow-through aquaria of the National Sea Simulator at the Australian Institute of Marine Science (AIMS), Townsville. Samples were collected under permit from the Great Barrier Reef Marine Park Authority issued to the Australian Institute of Marine Science. Five days post-collection, corals were cut into nubbins (*P*. *speciosa*: discs of ~5 cm diameter, *A*. *millepora*: three to five branches/nubbin ~5 cm tall). Colony identity of each nubbin was recorded to account for differences between colonies [15]. A total of 64 nubbins per species (n = 8 per parental colony) were glued to labeled ceramic plugs and placed in indoor flow-through holding tanks for a three-week acclimation and recovery period at controlled temperature (25.0°C, which corresponded the temperature at Davies Reef at the time of collection) and light regime (~7.5 mol photons m^-2^ d^-1^, light ramping up over six hours, a one hour at maximum irradiance of 250–350 μmol photons m^-2^ s^-1^, then 6 h light ramping down, and 11 h darkness). Due to logistical constraints, pre-experimental light conditions were lower than ideal and the subsequent transition into the high-light conditions for the experiment may have induced additional stress.

For the 20-days experiment, four nubbins per species, each from a different colony, were placed in each of sixteen 110 L aquaria. Each aquaria was equipped with a pump for circulation, and had a water exchange rate of 800 mL min^-1^. Water temperature was kept well below bleaching thresholds, between 25.0–26.0°C. Tanks in high-light treatments were, on average, 0.5°C warmer during periods of noontime irradiance than those in low-light; such temperature differences due to sunlight are not uncommon in shallow reef environments (e.g. [34]). Tanks were cleaned every two days to minimize algal growth. Corals were fed *Artemia* at concentrations of 0.5 nauplii mL^-1^, five times per week, two hours prior to ‘sunrise’.

Four DLI treatments (four tanks per treatment) were established, using Hydra LED lamps (Aquaillumination, USA) above each tank that were controlled to follow the ramping as outlined above with an extended noontime to emphasize differences between treatments. DLI treatments consisted of: high-light (HL: noontime irradiance 750–850 μmol photons m^-2^ s^-1^, corresponding to ~32 mol photons m^-2^ d^-1^), low-light (LL: noontime irradiance 125–175 μmol photons m^-2^ s^-1^, ~6 mol photons m^-2^ d^-1^), and two variable light groups (VL1 and VL2). The variable light treatments systematically alternated between 4 days of HL and LL, with each transition containing one additional day of intermediate light. VL1 was first exposed to LL conditions, whereas VL2 was first exposed to HL. VL1 and VL2 corals experienced on average ~17 mol photons m^-2^ d^-1^. Treatments reflected the magnitude of daily variation in light (~5 fold) and light extremes experienced by corals on shallow reefs during summer months (see examples [19, 21, 35–37], and [5] for a compilation), and the experimental daily integrated light levels and maximum noon irradiance were within the range of what corals naturally experience at Davies Reef (see S2 Fig). Black plastic sheets placed between tanks limited light spillover between treatments.

### Measurements of coral photoacclimation

Four sets of photoacclimation and physiological responses were measured as outlined in the sections below. They included (i) changes in photosynthetic potential and non-photochemical quenching, (ii) photosynthetic and photoprotective pigment content of the algal symbionts, (iii) photosynthesis to irradiance curves based on oxygen respirometry to understand the diurnal dynamics of photosynthesis and respiration [38], and (iv) buoyant weight change in *A*. *millepora* to measure growth [39, 40]. These approaches were chosen for consistency with the literature and to target symbiont responses (PAM and pigment quantification), to understand effects at the whole-colony level (respirometry) and to link to demographic rates (growth).

#### Symbiont photobiology measured using chlorophyll fluorescence

Fluorescence measurements were conducted using a diving pulse-amplitude modulated fluorometer (DPAM; Walz, Germany) [41], with a consistent distance between fiber optic tip and coral tissue, and standard settings (measuring intensity = 8, saturation intensity = 8, saturation width = 0.6 s, gain = 2, damping = 2). Each day (excluding ramp days) measurements were taken twice with the DPAM: once at 0.5 h before sunrise, to assess the maximum quantum yield of photosystem II (F_v_/F_m_), and at noon, after 0.5 h exposure to maximum irradiance, to assess effective quantum yield (PSIIϕ). F_v_/F_m_ represents the maximum potential for photosynthesis through quantification of ‘open’ photosystems (fully relaxed and oxidized, ready to process photon energy), and can be a useful proxy for photodamage [41]. Reductions in F_v_/F_m_ suggest that the rate of repair of PSII is too slow to keep up with the damage, and hence can show photodamage over time [42]. For each nubbin, at least five measurements were taken from different regions on each nubbin and the values averaged. Nubbins were split into two groups (n = 32 nubbins / group) based on colony ID and one group was measured daily, alternating groups each day. The excitation pressures on PSII, Q_m_ = 1 –(PSIIΦ / F_v_/F_m_) was assessed to estimate the degree of photoinhibition versus light limitation [29]. This measurement demonstrates the relative amount of open and closed versus available photosystems to give an estimation of the type of light stress (photoinhibitory vs. light-limited). High values of Q_m_ (above ~0.3) would indicate that the majority of available PSII reaction centers are closed at noontime irradiance levels (i.e. photoinhibition stress), whereas low values (~0) indicate most PSII reaction centers are open and not being utilized (light limitation). Non-photochemical quenching (NPQ), also derived from PAM pre-dawn and noontime measurements based on equations by Genty et al [43], was measured to assess the amount of excess photon energy dissipated safely as heat, and can act as a proxy for xanthophyll cycle activity [28, 44].

#### Symbiont pigment concentration via spectrophotometry

At the end of the experiment, the concentration of chlorophyll *a* (photosynthetic) and total carotenoids (photosynthetic and photoprotective) of nubbins were compared between treatments. Tissue was removed from the skeleton with an air gun and filtered seawater, and homogenized. The slurry was centrifuged for 6–8 min at 1,500 g and the coral host supernatant was separated from the symbiont pellet. The pellet was then rinsed with filtered seawater and re-centrifuged at 10,000 g for 3 min prior to extraction. Pigments were obtained via a double extraction procedure (1 mL 95% ethanol at 4°C for 20 minutes each, with sonicator), and the absorbance was spectrophotomerically measured at 665, 664, 649 and 470 nm wavelengths. Concentrations of chlorophyll *a* and total carotenoids (μg/mL) were calculated based on equations by Lichtenthaler [45] and Ritchie [46] and standardized to nubbin surface area, which was estimated via a single wax dip protocol [47]. Chlorophyll *a* concentration demonstrates photosynthetic potential, whereas carotenoids can suggest a photoprotective capability when considered with up-regulation of NPQ [42].

#### Photosynthesis and respiration measured by oxygen-respirometry

At the end of the experiment, 18 nubbins were selected for respirometry measurements. Their ceramic plugs were carefully cleaned to remove algal growth. Nubbins were individually placed in 634 mL sealed stirred chambers that contained oxygen sensor spots (optodes), and the Firesting hardware/software (Pyroscience, Germany) was used to measure oxygen concentrations within the chambers every minute. Incubations ran for an hour each at ten light levels (0, 15, 40, 80, 120, 200, 300, 500, 700 and 1000 μmol photons m^-2^ s^-1^), measured with an upwards facing, calibrated, cosine correctedlight sensor (meter LI-250A, sensor LI-192, Li-COR, USA). Water was flushed in the chambers at the beginning of each light level measurement. Rates of oxygen consumption (estimated respiration in the dark) and production (estimated net photosynthesis in the light) were standardized to coral surface area estimates derived from the wax dipping procedure. Photosynthesis to irradiance (P-I) curves were fitted to the data using a hyperbolic tangent fit, as described by Jassby and Platt [38] using the ‘stats’ package (version 3.6.0) in the statistical platform R (version 3.4.0, R Development Core Team 2017). Parameters for maximum photosynthetic production (P_max_), saturation irradiance (I_k_) and dark respiration (R_dark_) for each treatment were estimated from fitted models. Net daily oxygen production (Pn) was calculated by predicting production using the P-I curves at actual logged experimental light levels, over a 24 h period. Net oxygen production acts as a proxy for daily net photosynthetic production and gives an indication of potential energy reserves [48].

#### Colony growth rate

Growth rates of *A*. *millepora* were assessed as differences in buoyant weight over time [40]. Nubbins were individually weighed to the nearest 0.001 g by suspending them on a tray below a semi-micro balance (Shimadzu AUW220D, Japan) in a water bath at ~25 ^O^C. The percent change in buoyant weight between days 8 and 20 was assessed. Growth data were unavailable for *P*. *speciosa* as its slow growth prevents measurable changes in buoyant weight over the time scale of the experiment.

### Data analysis

Analyses were performed for each species separately in the statistics platform R (version 3.4.0, R Development Core Team 2017). All data were tested for normality and homogeneity of variance, and growth and pigment data were square-root transformed prior to analysis. Changes in F_v_/F_m_ and Q_m_ over time were assessed for each treatment separately using general additive mixed effects models (GAMM) as individual nubbins were measured repeatedly over time and are not independent. The ‘mgcv’ package (version 1.8–18) in R was used, with models assessing the PAM variable over time for each treatment including fragment identity as random effect. Significant changes (positive or negative) for the full 20 days in the constant light treatments indicated how long corals took to acclimate (denoted as stabilization of F_v_/F_m_), and changes in photoinhibition/light-limitation stress over time (Q_m_). In the variable light treatments, significant changes in the measured variables were assumed to represent active photoacclimation and/or photodamage.

To determine whether corals acclimate towards the average DLI, the minimum/maximum DLI, or continually adjusted as DLI changed, comparisons of F_v_/F_m_ between treatments were made at on days 5, 10, 15 and 20 (i.e., after four days exposure to unchanged light levels for the variable treatments) via one-way ANOVAs to assess the relative photoacclimatory state in each treatment. A Bonferroni correction was applied to account for these multiple comparisons.

To determine changes in photosynthetic potential and photoprotective xanthophyll cycling in response to changing light conditions, GAMMs were also applied to F_v_/F_m_ and NPQ data over the three transition periods (a six-day period including the final day of one segment, the day with intermediate light, and all four days of the following segment) (Bonferroni correction also applied). To assess rates of acclimation during the three transition events, the change in F_v_/F_m_ (slope) for each colony was compared between treatments. F_v_/F_m_ data during the transition periods were approximately linear, so linear mixed effects models (GLMM) were used to assess whether corals acclimated within this time frame (significant slopes represent active adjustment). Models included fragment identity as random effect and were run using the ‘lme4’ package (version 1.1–17) in R. To assess how quickly acclimation occurred (acclimation coefficient ε = ΔF_v_/F_m_ d^-1^), the absolute value of the slopes (|ε|) at the end of each five-day segment was compared using a one-way repeated measures ANOVA (rmANOVA—as data points were not independent) to determine if |ε| decreased or increased over time. |ε| was also compared between light conditions using a Wilcoxon Signed Rank test to determine if acclimation rates were equal when going into high versus low-light conditions.

To investigate whether light stress (Q_m_) decreased throughout the experiment, Q_m_ data were compared between days 5, 10, 15 and 20 using one-way rmANOVAs, separately for constant high-light and low-light conditions. For variable light treatments, data were divided into high and low-light events to test if stress levels (photoinhibition and light-limitation) changed over time.

Pigment concentrations of *A*. *millepora* were compared between treatments using two-way ANOVA, also testing for variation due to colony identity. For *P*. *speciosa*, one-way ANOVA was used (too few replicates per treatment were available to consider this factor statistically in this species, due to difficulties separating tissue from skeleton). Tukey HSD post-hoc analyses were run for both species where there was no interaction effect. Differences in I_k_, P_max_, R_dark_ and Pn between treatments were assessed using one-way ANOVAs. Finally, to investigate relative impact of variable versus constant light on growth in *A*. *millepora*, a one-way ANOVA with Tukey HSD post-hoc test was used to compare percent buoyant weight changes between treatments.

## Results

### Photosynthetic potential of corals (maximum quantum yield, F_v_/F_m_)

**Fig 1:**
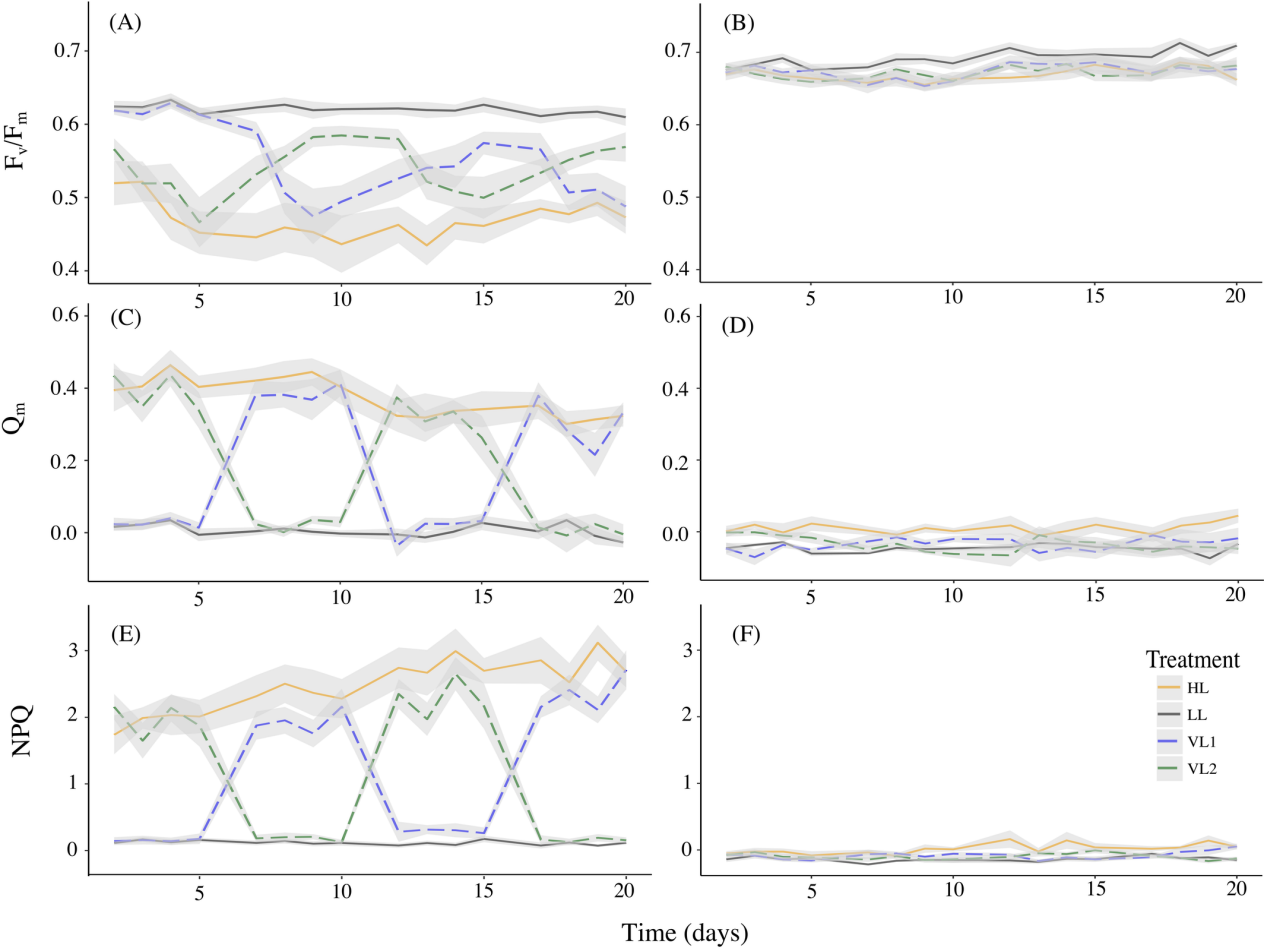
Photosynthetic potential, light stress and non-photochemical quenching under constant high and low DLI, and under two variable DLI treatments. Mean maximum quantum yield (F_v_/F_m_), excitation pressure on PSII (Q_m_) and non-photochemical quenching (NPQ) of *Pachyseris speciosa* (A, C, E)—and *Acropora millepora* (B, D, F), over the 20-days experiment in high DLI treatment (HL, orange), low DLI treatment (LL, purple), variable DLI 1 treatment (VL1, blue dashed lines) and variable DLI 2 treatment (VL2, green dashed lines). Values represent means over 16 colonies per treatment per species, with shaded areas representing standard error.

In *Pachyseris speciosa*, maximum quantum yield (F_v_/F_m_) decreased in the constant high DLI treatment (HL) during the first five days, then stabilized and began to increase during the second half of the experiment (GAMM edf = 2, F = 9.091, P < 0.001; Fig 1A). F_v_/F_m_ in the low DLI treatment (LL) remained stable (GAMM edf = 1, F = 1.732, P = 0.191). In variable DLI treatments, F_v_/F_m_ consistently tracked the levels of light, approaching values found within the constant light counterparts at the third to fifth day of all four segments (Fig 1 and S1 Table). F_v_/F_m_ in the variable DLI treatments were on average 0.59 ± 0.008 SE by the end of the low DLI segments (~5% less than the average LL values of 0.62 ± 0.005 SE), whereas by the end of high DLI segments, they averaged 0.49 ±0.014 SE (6.5% above of the average HL value of 0.46 ± 0.014 SE).

In *Acropora millepora*, F_v_/F_m_ in HL remained stable (GAMM edf = 1, F = 0.545, P = 0.462, Fig 1B), whereas in LL there was a slight but steady increase (~3.5%) over 20 days (GAMM edf = 1, F = 9.932, P < 0.01). In VL1 and VL2, F_v_/F_m_ of *A*. *millepora* averaged 0.67 ± 0.0025 SE and 0.67 ± 0.0019 SE, respectively, throughout the 20-day experiment, similar to the value in the HL constant treatment (0.662 ± 0.0086) and remained constant throughout the experiment (GAMM, P > 0.05). There were no significant differences in F_v_/F_m_ between the four treatments by the end of each segment for *A*. *millepora* (S1 Table), although LL had the greatest photosynthetic potential at day 20 (0.709 ± 0.0044 SE), 6.6% greater than in HL (0.662 ± 0.0086 SE) and 4% more than the average of VL1 and VL2 (0.68 ± 0.0097 SE).

### Rate of transition between light environments in variable light

*P*. *speciosa* demonstrated significant changes in F_v_/F_m_ within one day after changing DLI in both variable light groups (Table 1). Comparisons of the acclimation rate (ε) between the three transitions demonstrated that the magnitude of change during the first transition (0.036 ΔF_v_/F_m_ d^-1^ ± 0.007 SE) was most pronounced, approximately 50% and 70% greater than transitions two (0.023 ΔF_v_/F_m_ d^-1^ ± 0.003 SE) and three (0.021 ΔF_v_/F_m_ d^-1^ ± 0.003 SE), respectively (rmANOVA F_1,33_ = 15.203, P < 0.001). ε was on average -0.022 ΔF_v_/F_m_ d^-1^ ± 0.002 SE when transitioning into high DLI and 0.018 ΔF_v_/F_m_ d^-1^ ± 0.002 SE when transitioning into low DLI, without statistical difference between the absolute values of ε going either way (WSR V = 83, P = 0.165).

**Table 1:** Analysis of the extent of change in F_v_F_m_ and NPQ after transitioning between high and low levels of light.

Species	Treat-ment	Para-meter	Transition	Edf	F-value	P-value
*P*. *speciosa*	VL1	F_v_F_m_	1 (LL→HL)	1.00	35.05	< 0.001
			2 (HL→LL)	1.66	24.39	< 0.001
			3 (LL→HL)	1.86	26.63	< 0.001
		NPQ	1 (LL→HL)	1.99	147.90	< 0.001
			2 (HL→LL)	1.99	93.96	< 0.001
			3 (LL→HL)	1.98	90.17	< 0.001
	VL2	F_v_F_m_	1 (HL→LL)	1.68	42.21	< 0.001
			2 (LL→HL)	1.00	43.05	< 0.001
			3 (HL→LL)	1.00	14.94	< 0.001
		NPQ	1 (HL→LL)	1.98	101.20	< 0.001
			2 (LL→HL)	1.99	141.50	< 0.001
			3 (HL→LL)	1.99	115.20	< 0.001
*A*. *millepora*	VL1	F_v_F_m_	1 (LL→HL)	1.00	4.03	0.052
			2 (HL→LL)	1.51	3.75	0.045
			3 (LL→HL)	1.00	0.19	0.667
		NPQ	1 (LL→HL)	1.00	2.59	0.116
			2 (HL→LL)	1.00	5.81	0.021
			3 (LL→HL)	1.00	10.09	< 0.016
	VL2	F_v_F_m_	1 (HL→LL)	1.88	4.31	0.023
			2 (LL→HL)	1.61	1.55	0.217
			3 (HL→LL)	1.00	5.52	0.024
		NPQ	1 (HL→LL)	1.00	0.26	0.610
			2 (LL→HL)	1.00	6.10	0.018
			3 (HL→LL)	1.42	9.40	< 0.016

General additive mixed effects model result summaries for both variable light treatments (VL1 and VL2): change in maximum quantum yield (F_v_/F_m_) and non-photochemical quenching (NPQ) during transitions 1, 2 and 3 for *Pachyseris speciosa and Acropora millepora*. N = 5/transition/species. Critical P-value with Bonferroni correction α/3 = 0.016.

On average, F_v_/F_m_ of *A*. *millepora* did not change significantly during the transition between high and low DLI (Table 1), although six nubbins (14%) showed significant change (S2 Table). On average, ε of *A*. *millepora* was 0.003 ΔF_v_/F_m_ d^-1^ ± 0.0009 SE when transferring into low DLI, and -0.00002 ΔF_v_/F_m_ d^-1^ ± 0.0008 SE when transferring into high DLI. There was no significant difference in the magnitude of ε between transitions (rmANOVA F_1,34_ = 0.362, P = 0.551), nor between transition from high to low and vice versa (WSR V = 146, P = 0.3038).

### Energy dissipation–non-photochemical quenching

Levels of NPQ under constant HL gradually increased over the course of the experiment for both *P*. *speciosa* (GAMM edf = 1.013, F = 16.1, P < 0.0001) and *A*. *millepora* (GAMM edf = 1, F = 6.542, P < 0.05), although, for *P*. *speciosa*, NPQ increased ten times more than for *A*. *millepora* (Fig 1E and 1F). Under constant LL, NPQ did not change for *P*. *speciosa* (GAMM edf = 1, F = 2.066, P = 0.153) or for *A*. *millepora* (GAMM edf = 1.669, F = 0.672, P = 0.378).

In variable DLI, *P*. *speciosa* showed significant up-regulation of NPQ the day after transitions into high DLI, and down-regulation after transition to low DLI (Table 1). On average, levels of NPQ during high DLI were ~10-fold greater than those in low DLI (2.131 ± 0.06 SE vs 0.195 ± 0.02 SE). *A*. *millepora* showed only significant up/down-regulation of NPQ during the final transition. Levels remained similarly low in both treatments throughout the experiment (on average -0.065 ± 0.11 SE and -0.12 ± 0.09 SE for high and low DLI episodes, respectively).

### Light stress proxy—excitation pressure on PSII

For *P*. *speciosa* in HL, the excitation pressure on PSII (Q_m_) decreased steadily during the experiment (GAMM edf = 1, F = 11.84, P < 0.001; Fig 1C), from 0.39 ± 0.0632 SE on day 2 (suggesting light inhibition) to 0.32 ± 0.0293 SE on day 20 (8% decrease). In LL, Q_m_ remained stable (GAMM edf = 1, F = 2.76, P = 0.099), demonstrating chronic light-limitation (on average 0.006 ± 0.004 SE throughout the experiment). Under variable DLI, Q_m_ alternated between photoinhibited (0.35 ± 0.011 SE) and light limited states (0.016 ± 0.005 SE) within a day of changing DLI. During low DLI segments (i.e. light limiting conditions), the degree of light stress did not change over time (rmANOVA F_1,14_ = 0.151, P = 0.704), remaining on average 0.018 ± 0.01 SE throughout the experiment. During the high-light segments (i.e. photoinhibiting conditions), there was a significant decrease in Q_m_ the second time *P*. *speciosa* nubbins were exposed to high-light (rmANOVA F_1,14_ = 17.148, P < 0.001); Q_m_ was on average 0.38 ± 0.03 SE by the end of each high DLI segment during the first phase of the experiment (days 5 and 10), then dropped ~20% to 0.3 ± 0.03 SE during the second phase (days 15 and 20).

For *A*. *millepora*, Q_m_ did not change throughout the experiment in any of the treatments (GAMMs, all P > 0.1; Fig 1D), suggesting chronic light-limitation under all conditions, with negative values potentially indicating occurrence of chlororespiration (i.e. an alternative electron transport chain providing symbionts with an additional inorganic carbon source).

### Photosynthetic and photoprotective pigmentation

**Fig 2:**
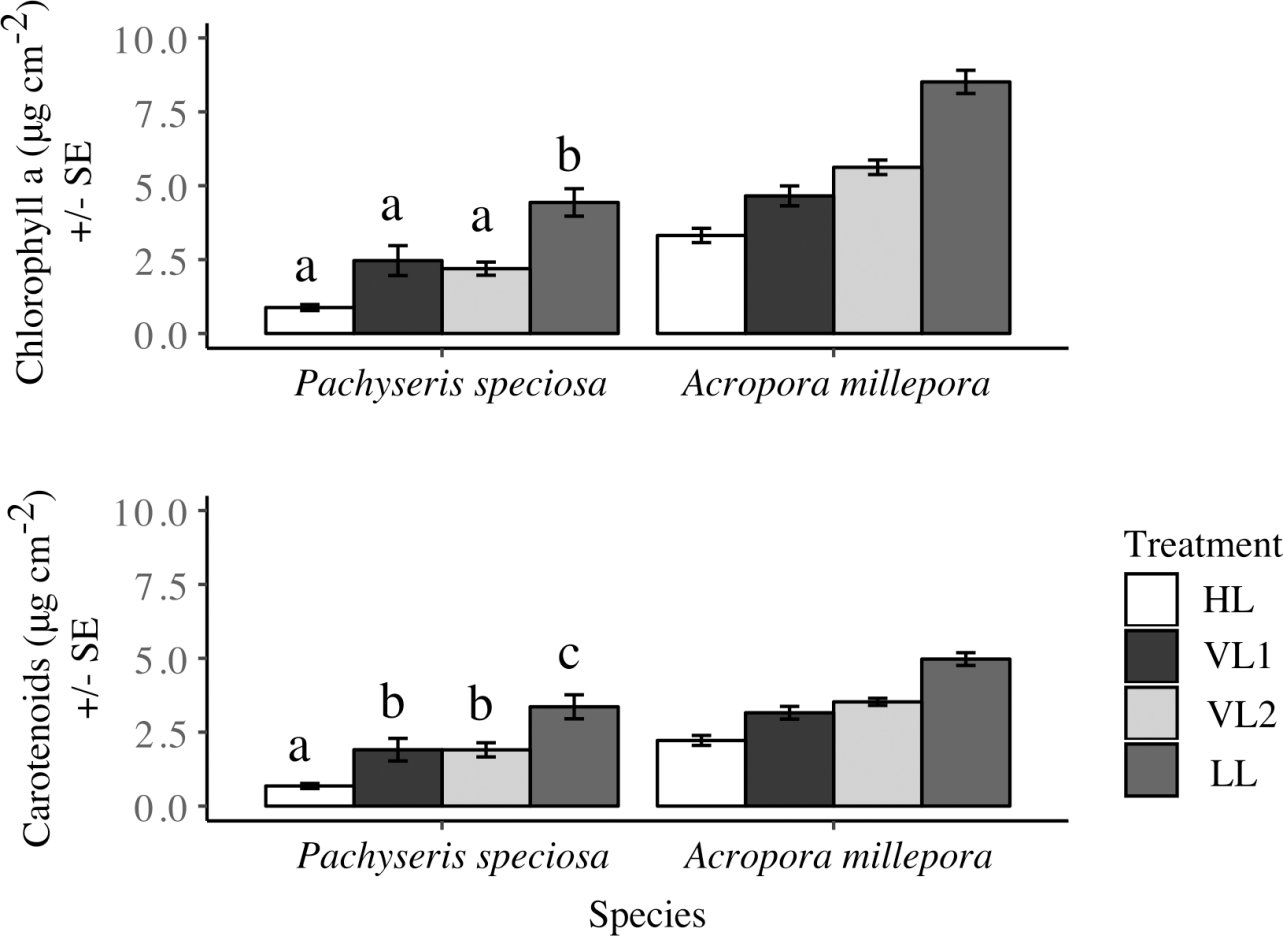
Effects of constant and variable light on pigment concentrations. Concentration of chlorophyll a (μg cm^-2^) and total carotenoids (μg cm^-2^) in *Pachyseris speciosa* (N = 5–6 nubbins/treatment) and *Acropora millepora* (N = 16 nubbins/treatment) under high DLI (white), low DLI (black), and variable DLI (VL1, light gray, and VL2, dark gray) treatments at the end of the 20-days experiment. Tukey HSD post-hoc results from one-way ANOVA comparison superimposed. Error bars represent standard error.

Concentrations of chlorophyll a and total carotenoids were highest in LL and lowest in HL in both species (Fig 2). For *P*. *speciosa*, both chlorophyll a and total carotenoid concentrations in the variable DLI were ~50% and ~40% lower than in LL, but more than double compared with HL treatments (ANOVA for chlorophyll a; F_3,17_ = 10.59, P < 0.0001 and total carotenoids; F_3,17_ = 14.82, P < 0.0001).

In *A*. *millepora*, HL nubbins had generally less chlorophyll a and total carotenoids than those in LL, and variable DLI treatments had ~30% higher concentrations than HL and 40% and 32% less chlorophyll a and total carotenoids than LL, respectively. However, some colonies showed different magnitudes of change between treatments, resulting in a significant interaction between treatments and colony (ANOVA chlorophyll a F_20,35_ = 2.021, P < 0.05; total carotenoids F_20,35_ = 2.017, P < 0.05).

### Photosynthesis to irradiance curves and net daily production

**Fig 3:**
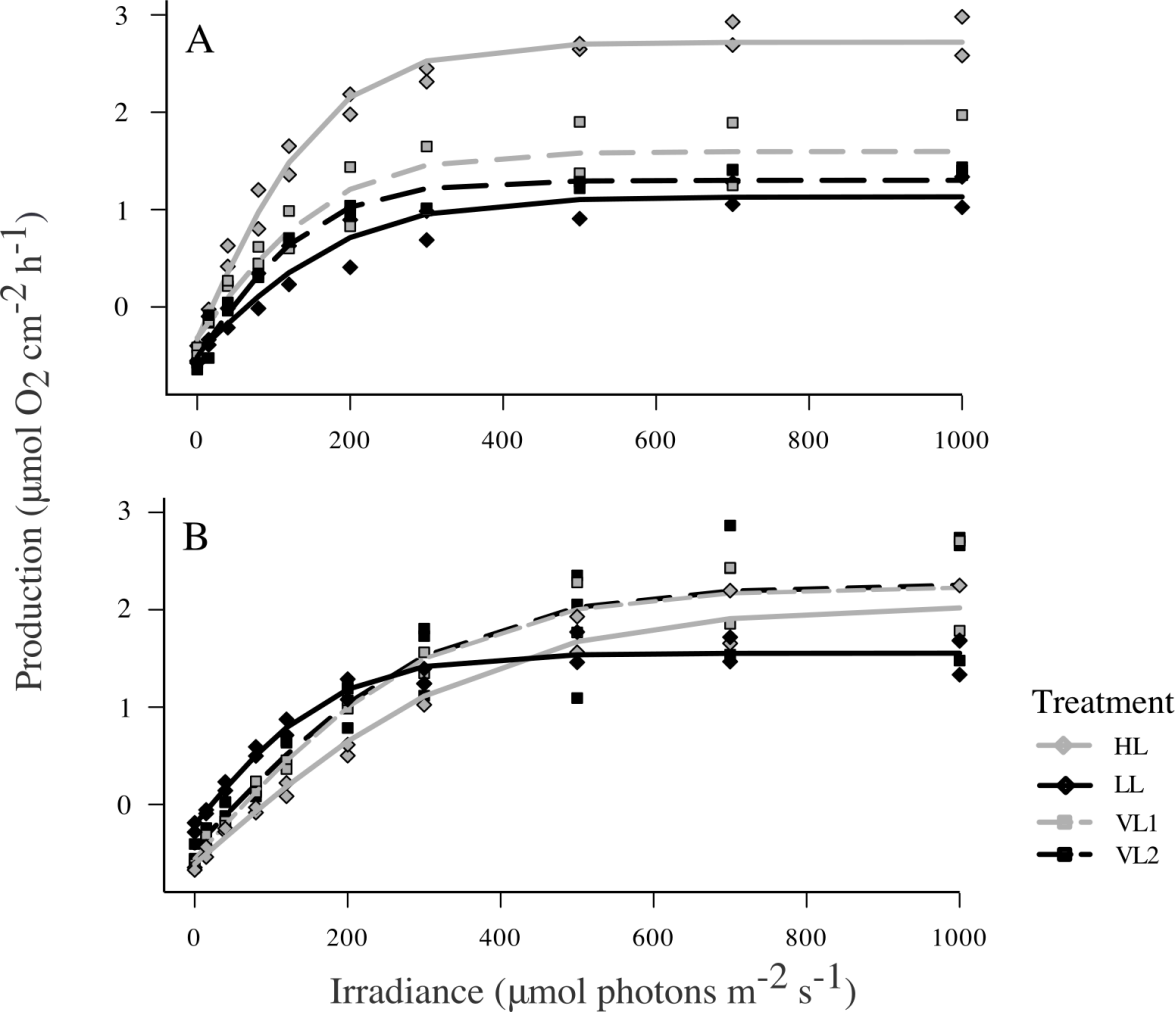
Photosynthesis-irradiance curves describing the contrasting photosynthetic features of the study species. Mean light-dependent oxygen production or consumption (μmol O_2_ cm^-2^ h^-1^) for *Pachyseris speciosa* (A) and *Acropora millepora* (B) at the end of the 20-day experiment in the high DLI (HL, solid grey), low DLI (LL, solid black), and variable DLI (VL1, dashed light gray, and VL2, dashed dark gray) treatments. N = 2–3 colonies/treatment/species.

**Fig 4:**
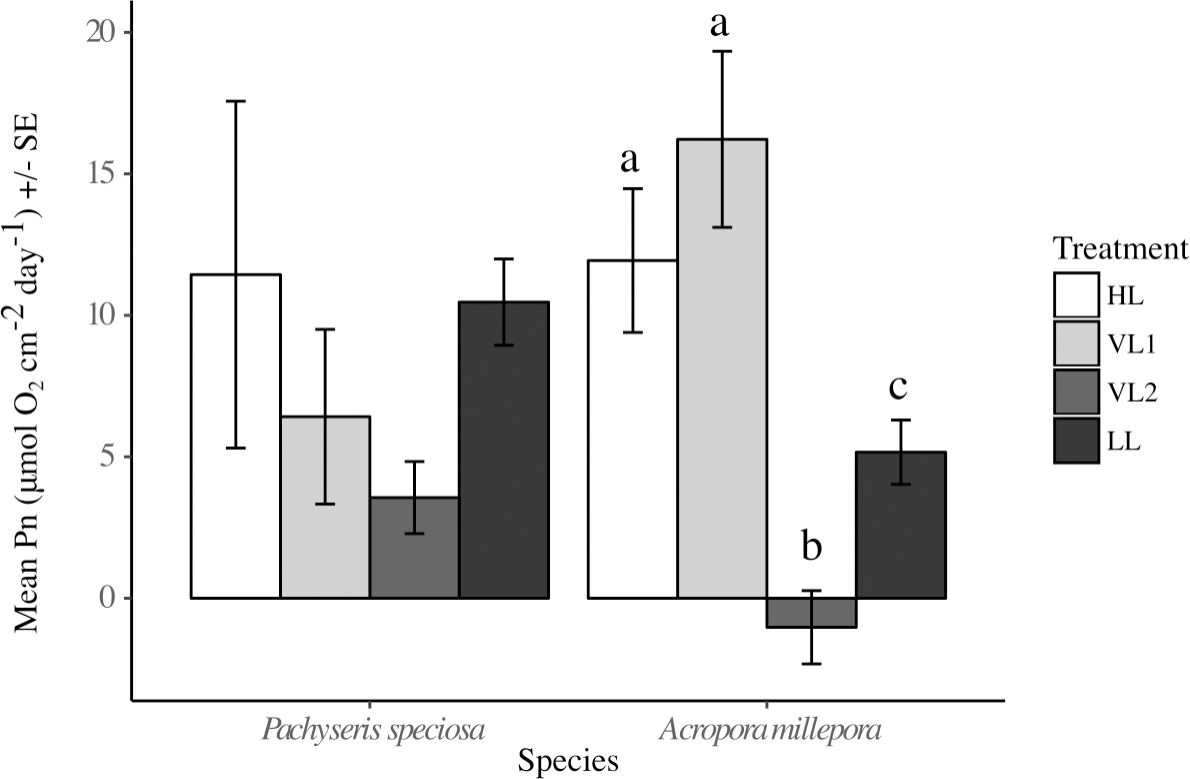
Effects of constant versus variable light on daily net oxygen production. Net daily production, Pn (μmol O_2_ cm^-2^ d^-1^), for *Pachyseris speciosa* and *Acropora millepora* in high DLI (HL, white), low DLI (LL, black), variable DLI treatment ending in high-light (VL1, light gray) and variable DLI treatment ending in low-light (VL2, dark gray) derived from P-I curves. Error bars represent standard error, N = 2-3/treatment/species.

No significant photoinhibition was observed for either species of coral when generating the photosynthesis to irradiance curves (P-I curves). Parameters derived from P-I curves, namely maximum photosynthetic production (P_max_), saturation irradiance (I_k_), dark respiration (R_dark_), and daily net production (Pn) did not greatly differ between the four treatments for *P*. *speciosa* (Fig 3A). LL had the greatest P_max_ (3.1 μmol O_2_ cm^-2^ h^-1^ ± 0.09 SE), almost twice that of HL (1.6 μmol O_2_ cm^-2^ h^-1^ ± 0.07 SE), and 30% and 37% more than VL1 (2.2 μmol O_2_ cm^-2^ h^-1^ ± 0.09 SE under low DLI) and VL2 (1.9 μmol O_2_ cm^-2^ h^-1^ ± 0.09 SE under high DLI), respectively (S3 Table), however differences were not statically significant (ANOVA F_3,5_ = 3.569, P = 0.102). I_k_ and R_dark_ values of variable light treatments were most similar to the corresponding constant light treatment, however again neither parameter was statistically different between treatments (ANOVA I_k_ F_3,5_ = 3.569, P = 0.102; R_dark_ F_3,5_ = 2.558, P = 0.168). Mean Pn in HL was almost three times of that in LL, and Pn in the variable treatments were in between, but variability was high and values did not differ statistically between treatments (ANOVA F_3,5_ = 0.98, P = 0.472; Fig 4).

*A*. *millepora* showed similar P-I curves in the HL and variable treatments (Fig 3B), and mean P_max_ in these three treatments varied only between 2.6 and 2.9 μmol O_2_ cm^-2^ h^-1^. In contrast, the LL group showed a characteristic low-light P-I curve, with lower P_max_ (1.8 μmol O_2_ cm^-2^ h^-1^ ± 0.05 SE), while I_k_ (387.3 μmol photons m^-2^ s^-1^ ± 28.5 SE) was on average ~40–60% lower than the other treatments, although no significant differences were found (ANOVA P_max_ F_3,5_ = 1.647, P = 0.292; I_k_ F_3,5_ = 4.109, P = 0.0811). The LL group had significantly lower dark respiration than all other treatments (ANOVA R_dark_ F_3,5_ = 26.71, P < 0.01). *A*. *millepora* corals under the variable treatment ending in low DLI (VL2) was the only one treatment demonstrating consistent and significant negative Pn (ANOVA, F_3,5_ = 15.53, P < 0.01; Fig 4).

### Relative colony growth (buoyant weight change of *A*. *millepora*)

**Fig 5:**
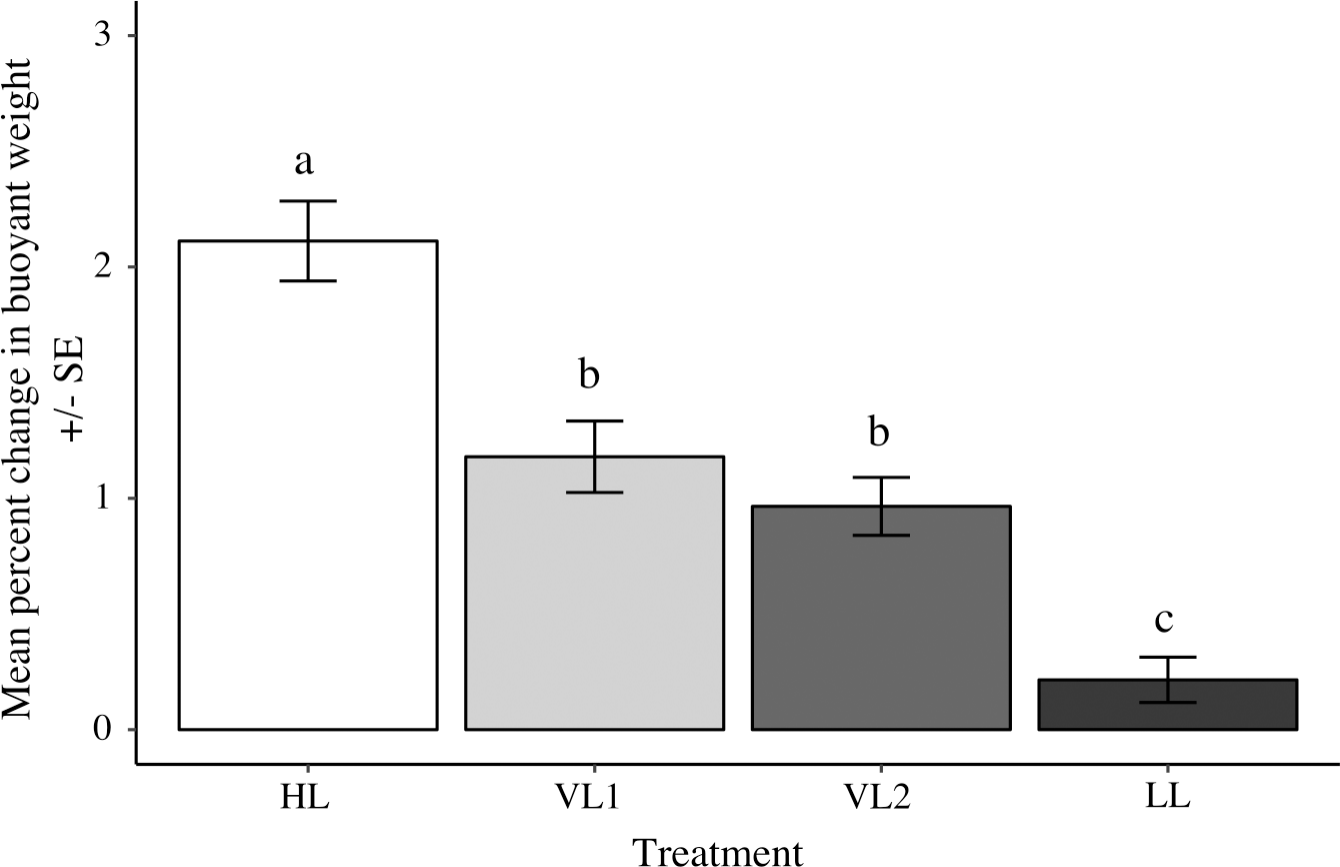
Relative growth of *Acropora millepora* between constant and variable DLI treatments. Mean percent change of *Acropora millepora* buoyant weight in the high DLI (HL, white), low DLI (LL, black), and two variable DLI treatments (VL1, light grey & VL2, dark grey) after 12 days. Tukey HSD post-hoc results from one-way ANOVA comparisons superimposed. Error bars represent standard error, n = 16 per treatment.

*A*. *millepora* in HL had significantly greater increases in buoyant weight (i.e., growth) after 12 days compared to corals in the other three treatment groups (ANOVA F_3,51_ = 33.3 P < 0.0001; Fig 5). Mean growth in HL was nearly 10 times greater than in LL (2.11% change d^-1^ ± 0.17 SE, vs. 0.22% d^-1^ ± 0.10 SE). Corals in the variable DLI treatments showed intermediate growth compared to LL and HL corals, with VL1 (1.18% change d^-1^ ± 0.15 SE) and VL2 (0.97% change d^-1^ ± 0.13 SE) growing 5.5 and 4.5 times faster than LL, respectively.

## Discussion

This study provides novel insights into photoacclimatory responses of reef corals to short-term (3–4 days) variation in daily light integrals (DLI), as would arise from changes in turbidity and cloud cover. The experiment showed that variable DLIs exerted physiological stress on both a low-light and a high-light tolerant coral species. The low-light coral *Pachyseris speciosa* photoacclimated to changing DLI within two days, with photoinhibition declining slightly under prolonged and repeated exposure to high DLI. At the colony level, oxygen production was relatively stable among the different constant and variable light treatments. In contrast, *Acropora millepora*, which is typically found in high-light environments, showed only minor and slow photoacclimatory responses, with limited oxygen production and growth under both low and variable DLI compared to under high DLI.

### Symbiont strategies for coping with variable DLI conditions

Symbionts within *P*. *speciosa* appear to adopt a photoacclimation strategy of continually and rapidly adjusting to new light environments. The observed immediate drop in F_v_/F_m_ when transitioning into high DLIs and decreased chlorophyll a pigmentation likely denotes a degree of photodamage and/or photoprotective dissociation of antenna complexes from PSII [49, 50], as previously observed in other corals that displayed F_v_/F_m_ levels of ~0.45 under high-light conditions [49, 51]. Low pigmentation in the high-light treatments likely indicates stress under high-light conditions, which is expected for the shade-adapted *P*. *speciosa*. Although F_v_/F_m_ was stable in both species under the high-light treatment, it is possible that repeated and/or continued exposure to high-light conditions would lead to bleaching and potentially even mortality, especially for *P*. *speciosa*. However, after several days in high DLI, *P*. *speciosa*’s gradual increase in F_v_/F_m_ and slow reduction in Q_m_ provides evidence of diminishing photoinhibition and suggests active acclimation to these conditions. Full acclimation, however, takes more than 4–5 days [29, 52], and hence adjustment of physiology to reach a steady-state was only observed in the constant high DLI and not in the variable treatments.

Importantly, the rapid onset of recovery towards near-baseline levels of F_v_/F_m_ [18] suggests that *P*. *speciosa* is quite resilient to short periods of high DLI exposure. The literature reports that other shade-adapted corals also demonstrate this immediate initiation of photorecovery following a shift in DLI levels, including deep water *Porites* [53], *Platygyra sinensis* and *P*. *speciosa* [18] and *Pavona* spp [54] in various short-term temperature and turbidity stress experiments. Such rapid recovery could be achieved through up-regulating mechanisms that dissipate excess light energy, such as the xanthophyll pigment cycle [55]. It is likely that pigments such as the xanthophylls and β-carotene may be the dominant carotenoids in high-light corals, as these pigments are known to act as photoprotectants so as to maintain higher concentrations of photosynthetic pigments [13, 16, 24]. Rapid up-regulation of NPQ during high-light episodes suggests that the xanthophylls were likely present and active in dissipating excess light energy [16, 28]. Increase in xanthophylls likely explains the intermediate concentration of carotenoids present in the variable treatments. The rapid recovery of photosynthetic potential is likely beneficial for corals such as *P*. *speciosa* in turbid, inshore Indo-Pacific reefs where significant declines in light availability are common events [18, 56, 57].

In contrast, *A*. *millepora* responded to variable DLI with few, minor and slow changes in their symbiont photoacclimatory responses, F_v_/F_m_ and pigmentation. *A*. *millepora* typically grows on the upper slopes and flats of reefs (2–5 m deep), where they can experience high-light exposure (>30 mol photons m^-2^ d^-1^, see S1 and S2 Figs), while their natural estimated minimum light threshold is ~5 mol photons m^-2^ d^-1^ [58]. The vertical alignment and dense spacing of branchlets in corymbose colony morphologies facilitate self-shading on all surfaces except the symbiont-free growing tips, reducing exposure to light [49, 59, 60]. Consistent with the habitat distribution of this species, our experiment showed that *A*. *millepora* experienced the greatest photosynthetic challenges under low DLI, and that acclimation took ≥20-days, meaning that the 5-day variations in light levels in the variable DLI treatments in this experiment are far shorter than the acclimation time for this species. This result is consistent with a previous study showing limited photoacclimation (to fixed light levels) over a 9-day experimental period for *A*. *millepora* and three other *Acropora* species [61]. Similarly, durations of a minimum of 10–20 days for short-term acclimation have been previously reported for *Turbinaria mesenterina* [7], although some other species, such as *Stylophora pistillata*, begin to acclimate within two to four days [23]. Moreover, between-colony variations in responses from both species suggest that additional physiological processes, such as host pigments and nutrient uptake, may significantly contribute to photoacclimation. These include accumulation of antioxidants and mycosporine-like amino acids [11, 62], green fluorescent proteins and GFP-like proteins [12, 30, 63], as well as tissue expansion/retraction [59] and differences in algal symbiont identity [64].

### Importance of light history on symbiont response

The observed trends in light stress experienced by *Pachyseris speciosa* (as measured by Q_m_), the degree of up-regulation of photoprotective NPQ, and rates of acclimation, all suggest that light history plays an important role in determining coral responses to fluctuating DLI. Our results showed that *P*. *speciosa* experienced lower photoinhibitory stress over repeated cycles of high DLIs, balancing declining photosynthetic pigments and increasing reliance on light-energy dissipation, for example through NPQ and intermediate carotenoid concentrations. Similar effects of light history on photoacclimation and NPQ are seen for short-term light changes on the scale of seconds to minutes [55, 65], however this is the first study that shows this influence on a larger scale (days).

Interestingly, there was no significant improvement in the light-limitation stress (Q_m_) in either species under variable conditions. Q_m_ is known to remain consistently low in corals under light-limiting conditions [29, 51], suggesting that deeper-water corals rely on other strategies to cope with low-light in the long term. Alternatively, physiological adjustments to avoid light-limitation (e.g. increasing chlorophyll *a* content) might be limited by other processes (e.g. nitrogen availability [14]), or require more than 20 days time for acclimation (e.g. adjusting symbiont densities [23]).

### Effects of variable DLI on coral net oxygen production and growth

The results of this study demonstrate two contrasting photosynthetic responses of corals to variable DLI. *P*. *speciosa* in the present study seemed to have been able to maintain net oxygen production, potentially in part due to rapid photosynthetic adjustment to the changing light environment to optimize light harvest. This is corroborated by slightly reduced respiration rates seen in the low and variable low treatment corals; low respiration can conserve energy in light-limited environments [21, 66]. Organic carbon and energy produced by photosynthesis can be utilized to build and repair proteins that are essential to mitigate photoinhibition, for pigment upregulation and/or for photosystem repair [39, 55, 67] and hence being able to maintain positive net oxygen production could be a valuable asset in variable light conditions. In contrast, variable DLI is detrimental via lowered generation of photosynthetic production during low DLI episodes for high-light tolerant corals such as *A*. *millepora* that are unable to rapidly adjust their photophysiology and conserve energy with reduced respiration rates [21, 62, 63]. Intermediate growth rates in variable DLI and low growth in low DLI further demonstrate the costs of living under low DLI. Our results also support the theory of light enhanced calcification, wherein increased photosynthesis at high-light directly enhances colony calcification and, thereby, reef accretion, by providing inorganic carbon and metabolic energy [68, 69]. It is important to note the 0.5°C warming during noon in the high-light treatments may have to a small degree further co-contributed to the faster growth observed under high-light. Further investigations into tissue composition, use of heterotrophic feeding as a buffer, and reproductive ability under variable DLIs are needed to fully understand the effects of increasing variability in the natural environment on coral energy budgets and overall fitness.

*A*. *millepora*’s slower growth under low and variable DLI and slow rates of symbiont photoacclimation have implications for its ability to endure in environments with fluctuating low-light availability. Inshore and even mid- and outer-shelf regions along parts of the GBR have experienced a distinct decrease in mean water clarity over the past decade or so [70–72]. These regions are exposed to terrigenous sediments via flood plumes and repeated wind driven resuspension [71, 72], leading to increased frequency and degree of reduction in benthic DLIs. Water clarity has been shown to have significant impact on coral health [73] and sufficient light availability can increase resiliency of corals under high sedimentation conditions [74], demonstrating the importance of light availability to corals when coping with stress. The cumulative effects of variable and low-light on coral net photosynthetic production and growth, as demonstrated in this study, suggest potentially negative implications for rates of reef growth and recovery.

## Conclusion

This study documented two different mechanisms through which variable light can affect corals with contrasting morphologies and photophysiologies. Firstly, the rapid recovery of photosynthetic potential and maintenance of positive net daily production under low DLI allows *Pachyseris speciosa* to quickly adjust to low DLI and to survive in turbid reef environments, however the rapid declines in photosynthetic potential demonstrates acute vulnerability to high DLI. In contrast, *Acropora millepora*’s photoprotective branching morphology enables it to tolerate high DLI, whereas it’s slow photoacclimation, inability to rapidly reduce energetically expensive respiration rates and significant growth reductions under variable and low DLI put this species at a disadvantage. This is especially relevant in the context of the globally increasing exposure of coral reefs to coastal runoff and dredging, which lead to increasing variability in water clarity and hence DLI.

## Supporting information

S1 Table Analysis of photosynthetic potential between treatments at four time points. ANOVA summaries comparing maximum quantum yield (F_v_/F_m_) between treatments on day 5, 10, 15 and 20 for *Pachyseris speciosa* and *Acropora millepora*. N = 16/treatment/species. Critical P-value with Bonferroni correction α/4 = 0.0125.
(DOCX)

S2 Table LMM model summary for each nubbin/colony during transition periods. Change in maximum quantum yield (ΔF_v_/F_m_) and corresponding p-value from linear mixed effects models for each coral nubbin (ID) of both *Pachyseris speciosa* and *Acropora millepora* in variable light treatments (VL1 and VL2) during all three transition events. * denotes significance. N = 6/nubbin.
(DOCX)

S3 Table Photosynthetic characteristics derived from P-I curves. Parameters of maximum photosynthetic potential (P_max_ μmol O_2_ cm^-2^ h^-1^), saturation irradiance (I_k_ μmol photons m^-2^ s^-1^) and dark respiration rates (R_dark_ μmol O_2_ cm^-2^ h^-1^), derived from photosynthesis-irradiance curves for *Pachyseris speciosa* and *Acropora millepora* between treatments, with ending light condition italics next to treatment. Standard error in parentheses. N = 2-3/treatment/species.
(DOCX)

S1 Fig Light attenuation for Davies Reef, GBR, Australia. Irradiance (μmol photons m^-2^ s^-1^) at depth (m) profile at 11am for Davies Reef, central Great Barrier Reef, at the time of coral collection in July 2016. N = 3
(TIF)

S2 Fig PAR data at 0.8m for Davies Reef, GBR, Australia. Davies Reef (A) mean daily light integrals (mol photons m^-2^ d^-1^) for May 2011 to May 2012 at 0.8m and (B) instantaneous PAR (μmol photons m^-2^ s^-1^) over the day in January 2012, colours representing the different days of the month. Data obtained via the AIMS Weather Station Program at: https://apps.aims.gov.au/metadata/view/076c8641-6e72-4be7-9eb7-e21145cc6525 and specifically http://data.aims.gov.au/aimsrtds/datatool.xhtml?from=1980-01-01&thru=2018-06-25&channels=9272,9273.
(TIF)

S1 Dataset PAM, pigment, respirometry and growth datasets. (XLSX)
