# Supplementary material for: Effects of variability in daily light integrals on the photophysiology of the corals Pachyseris speciosa and Acropora millepora
Source: PLoS One. 2018 Sep 21;13(9):e0203882. doi: 10.1371/journal.pone.0203882 (PMC6150484; doi:10.1371/journal.pone.0203882)
Supplement: S1 Table — ANOVA summaries comparing maximum quantum yield (Fv/Fm) between treatments on day 5, 10, 15 and 20 for Pachyseris speciosa and Acropora millepora. N = 16/treatment/species. Critical P-value with Bonferroni correction α/4 = 0.0125. (DOCX) [file pone.0203882.s001.docx]

**S1 Table. Analysis of** **photosynthetic potential between treatments at four time points.**

| **Species** | **Day** | **df** | **F-stat** | **p-value** |
| --- | --- | --- | --- | --- |
| P.speciosa | 5 | 3,28 | 19.54 | < 0.0001* |
|  | 10 | 3,27 | 16.74 | < 0.0001* |
|  | 15 | 3,28 | 14.9 | < 0.0001* |
|  | 20 | 3,28 | 11.09 | < 0.0001* |
| A.millepora | 5 | 3,28 | 1.178 | 0.336 |
|  | 10 | 3,28 | 2.032 | 0.132 |
|  | 15 | 3,28 | 1.528 | 0.229 |
|  | 20 | 3,28 | 3.648 | 0.046 |
