# Supplementary material for: Effects of variability in daily light integrals on the photophysiology of the corals Pachyseris speciosa and Acropora millepora
Source: PLoS One. 2018 Sep 21;13(9):e0203882. doi: 10.1371/journal.pone.0203882 (PMC6150484; doi:10.1371/journal.pone.0203882)
Supplement: S2 Table — Change in maximum quantum yield (ΔFv/Fm) and corresponding p-value from linear mixed effects models for each coral nubbin (ID) of both Pachyseris speciosa and Acropora millepora in variable light treatments (VL1 and VL2) during all three transition events. * denotes significance. N = 6/nubbin. (DOCX) [file pone.0203882.s002.docx]

| **Treatment** | **Transition** | **Species** | **ID** | **ΔF_v_/F_m_** | **P-value** | **Species** | **ID** | **ΔF_v_/F_m_** | **P-value** |
| --- | --- | --- | --- | --- | --- | --- | --- | --- | --- |
| VL1 | 1 (LL🡪HL) | *P. speciosa* | P1 | -0.017 | < 0.01* | *A. millepora* | A1 | -0.003 | 0.152 |
| VL1 | 2 (HL🡪LL) | *P. speciosa* | P1 | 0.011 | < 0.001* | *A. millepora* | A1 | 0.008 | 0.072 |
| VL1 | 3 (LL🡪HL) | *P. speciosa* | P1 | -0.018 | < 0.001* | *A. millepora* | A1 | 0.002 | 0.702 |
| VL1 | 1 (LL🡪HL) | *P. speciosa* | P3 | -0.026 | < 0.001* | *A. millepora* | A3 | -0.005 | 0.155 |
| VL1 | 2 (HL🡪LL) | *P. speciosa* | P3 | 0.016 | < 0.001* | *A. millepora* | A3 | 0.006 | < 0.001* |
| VL1 | 3 (LL🡪HL) | *P. speciosa* | P3 | -0.013 | < 0.01* | *A. millepora* | A3 | -0.003 | 0.651 |
| VL1 | 1 (LL🡪HL) | *P. speciosa* | P4 | -0.011 | < 0.001* | *A. millepora* | A4 | 0.003 | 0.289 |
| VL1 | 2 (HL🡪LL) | *P. speciosa* | P4 | 0.005 | < 0.001* | *A. millepora* | A4 | 0.002 | 0.680 |
| VL1 | 3 (LL🡪HL) | *P. speciosa* | P4 | -0.010 | < 0.05* | *A. millepora* | A4 | 0.006 | 0.525 |
| VL1 | 1 (LL🡪HL) | *P. speciosa* | P5 | -0.035 | < 0.001* | *A. millepora* | A6 | -0.006 | < 0.001* |
| VL1 | 2 (HL🡪LL) | *P. speciosa* | P5 | 0.023 | < 0.001* | *A. millepora* | A6 | 0.003 | < 0.01* |
| VL1 | 3 (LL🡪HL) | *P. speciosa* | P5 | -0.021 | < 0.001* | *A. millepora* | A6 | -0.004 | 0.536 |
| VL1 | 1 (LL🡪HL) | *P. speciosa* | P6 | -0.044 | < 0.001* | *A. millepora* | A7 | -0.003 | 0.587 |
| VL1 | 2 (HL🡪LL) | *P. speciosa* | P6 | 0.038 | < 0.001* | *A. millepora* | A7 | -0.002 | 0.710 |
| VL1 | 3 (LL🡪HL) | *P. speciosa* | P6 | -0.040 | < 0.001* | *A. millepora* | A7 | -0.001 | 0.780 |
| VL1 | 1 (LL🡪HL) | *P. speciosa* | P7 | -0.029 | 0.243 | *A. millepora* | A10 | -0.004 | < 0.05* |
| VL1 | 2 (HL🡪LL) | *P. speciosa* | P7 | 0.011 | < 0.05* | *A. millepora* | A10 | 0.008 | 0.038 |
| VL1 | 3 (LL🡪HL) | *P. speciosa* | P7 | -0.017 | 0.084 | *A. millepora* | A10 | -0.001 | 0.697 |
| VL1 | 1 (LL🡪HL) | *P. speciosa* | P8 | -0.036 | < 0.001* | *A. millepora* | A9 | -0.003 | 0.082 |
| VL1 | 2 (HL🡪LL) | *P. speciosa* | P8 | 0.028 | < 0.001* | *A. millepora* | A9 | 0.003 | 0.191 |
| VL1 | 3 (LL🡪HL) | *P. speciosa* | P8 | -0.014 | < 0.001* | *A. millepora* | A9 | 0.006 | 0.602 |
| VL2 | 1 (HL🡪LL) | *P. speciosa* | P1 | 0.026 | < 0.001* | *A. millepora* | A1 | 0.008 | 0.067 |
| VL2 | 2 (LL🡪HL) | *P. speciosa* | P1 | -0.018 | < 0.05* | *A. millepora* | A1 | 0.000 | 0.975 |
| VL2 | 3 (HL🡪LL) | *P. speciosa* | P1 | 0.014 | < 0.05* | *A. millepora* | A1 | 0.007 | < 0.01* |
| VL2 | 1 (HL🡪LL) | *P. speciosa* | P3 | 0.011 | < 0.001* | *A. millepora* | A3 | -0.001 | 0.846 |
| VL2 | 2 (LL🡪HL) | *P. speciosa* | P3 | -0.016 | < 0.05* | *A. millepora* | A3 | 0.003 | 0.563 |
| VL2 | 3 (HL🡪LL) | *P. speciosa* | P3 | 0.007 | 0.235 | *A. millepora* | A3 | -0.001 | 0.703 |
| VL2 | 1 (HL🡪LL) | *P. speciosa* | P4 | 0.011 | < 0.001* | *A. millepora* | A4 | 0.004 | 0.099 |
| VL2 | 2 (LL🡪HL) | *P. speciosa* | P4 | 0.000 | 0.992 | *A. millepora* | A4 | -0.001 | 0.648 |
| VL2 | 3 (HL🡪LL) | *P. speciosa* | P4 | -0.004 | 0.509 | *A. millepora* | A4 | 0.006 | 0.160 |
| VL2 | 1 (HL🡪LL) | *P. speciosa* | P5 | 0.001 | < 0.001* | *A. millepora* | A6 | 0.003 | 0.373 |
| VL2 | 2 (LL🡪HL) | *P. speciosa* | P5 | -0.024 | < 0.001* | *A. millepora* | A6 | 0.003 | 0.501 |
| VL2 | 3 (HL🡪LL) | *P. speciosa* | P5 | 0.021 | < 0.001* | *A. millepora* | A6 | 0.001 | 0.732 |
| VL2 | 1 (HL🡪LL) | *P. speciosa* | P6 | 0.026 | < 0.001* | *A. millepora* | A7 | 0.001 | 0.818 |
| VL2 | 2 (LL🡪HL) | *P. speciosa* | P6 | -0.036 | < 0.001* | *A. millepora* | A7 | 0.001 | 0.916 |
| VL2 | 3 (HL🡪LL) | *P. speciosa* | P6 | 0.027 | < 0.001* | *A. millepora* | A7 | 0.005 | 0.054 |
| VL2 | 1 (HL🡪LL) | *P. speciosa* | P7 | 0.016 | < 0.001* | *A. millepora* | A10 | -0.004 | 0.312 |
| VL2 | 2 (LL🡪HL) | *P. speciosa* | P7 | -0.016 | < 0.001* | *A. millepora* | A10 | 0.007 | 0.356 |
| VL2 | 3 (HL🡪LL) | *P. speciosa* | P7 | 0.017 | < 0.01* | *A. millepora* | A10 | 0.003 | 0.088 |
| VL2 | 1 (HL🡪LL) | *P. speciosa* | P8 | 0.022 | < 0.001* | *A. millepora* | A9 | -0.007 | < 0.01* |
| VL2 | 2 (LL🡪HL) | *P. speciosa* | P8 | -0.019 | < 0.001* | *A. millepora* | A9 | 0.003 | 0.299 |
| VL2 | 3 (HL🡪LL) | *P. speciosa* | P8 | 0.012 | < 0.05* | *A. millepora* | A9 | 0.001 | 0.604 |

**S2 Table: LMM model summary for each nubbin/colony during transition periods**
