# Supplementary material for: Effects of variability in daily light integrals on the photophysiology of the corals Pachyseris speciosa and Acropora millepora
Source: PLoS One. 2018 Sep 21;13(9):e0203882. doi: 10.1371/journal.pone.0203882 (PMC6150484; doi:10.1371/journal.pone.0203882)
Supplement: S3 Table — Parameters of maximum photosynthetic potential (Pmax μmol O2 cm-2 h-1), saturation irradiance (Ik μmol photons m-2 s-1) and dark respiration rates (Rdark μmol O2 cm-2 h-1), derived from photosynthesis-irradiance curves for Pachyseris speciosa and Acropora millepora between treatments, with ending light condition italics next to treatment. Standard error in parentheses. N = 2-3/treatment/species. (DOCX) [file pone.0203882.s003.docx]

**S3 Table: Photosynthetic characteristics derived from P-I curves**

|  | **HL -*High-*** | **LL -*Low-*** | **VL1 -*High-*** | **VL2 -*Low-*** |
| --- | --- | --- | --- | --- |
| ***P. speciosa*** |  |  |  |  |
| P_max (μmol O2 cm-2 hr-1)_ | 1.6 (0.07) | 3.1 (0.09) | 2.2 (0.09) | 1.9 (0.09) |
| I_k (μmol photons m-2 s-1)_ | 219.2 (55.9) | 179.7 (43) | 220.2 (58.9) | 188.2 (14.2) |
| R_dark (μmol O2 cm-2 hr-1)_ | -0.5 (0.05) | -0.3 (0.08) | -0.5 (0.08) | -0.3 (0.08) |
| ***A. millepora*** |  |  |  |  |
| P_max (μmol O2 cm-2 hr-1)_ | 2.7 (0.06) | 1.8 (0.05) | 2.9 (0.06) | 2.6 (0.13) |
| I_k (μmol photons m-2 s-1)_ | 387.3 (28.5) | 186.8 (8.9) | 318.2 (60.9) | 316.6 (36.3) |
| R_dark (μmol O2 cm-2 hr-1)_ | -0.6 (0.04) | -0.2 (0.04) | -0.5 (0.04) | -0.5 (0.09) |
