# Supplementary figures and images for: Effects of variability in daily light integrals on the photophysiology of the corals Pachyseris speciosa and Acropora millepora
Source: PLoS One. 2018 Sep 21;13(9):e0203882. doi: 10.1371/journal.pone.0203882 (PMC6150484; doi:10.1371/journal.pone.0203882)

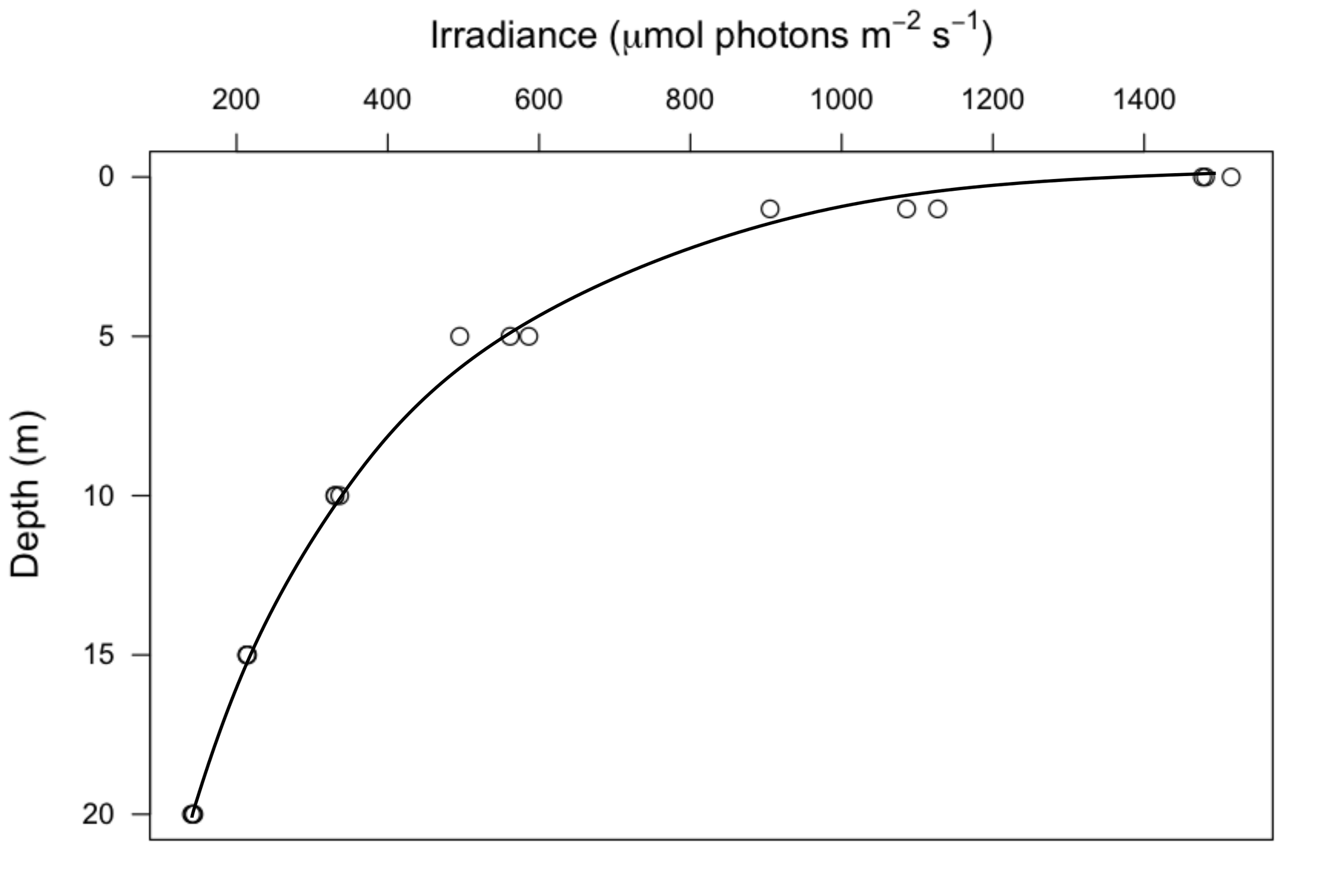

Supplement: S1 Fig — Irradiance (μmol photons m-2 s-1) at depth (m) profile at 11am for Davies Reef, central Great Barrier Reef, at the time of coral collection in July 2016. N = 3 (TIF) [file pone.0203882.s004.tif]

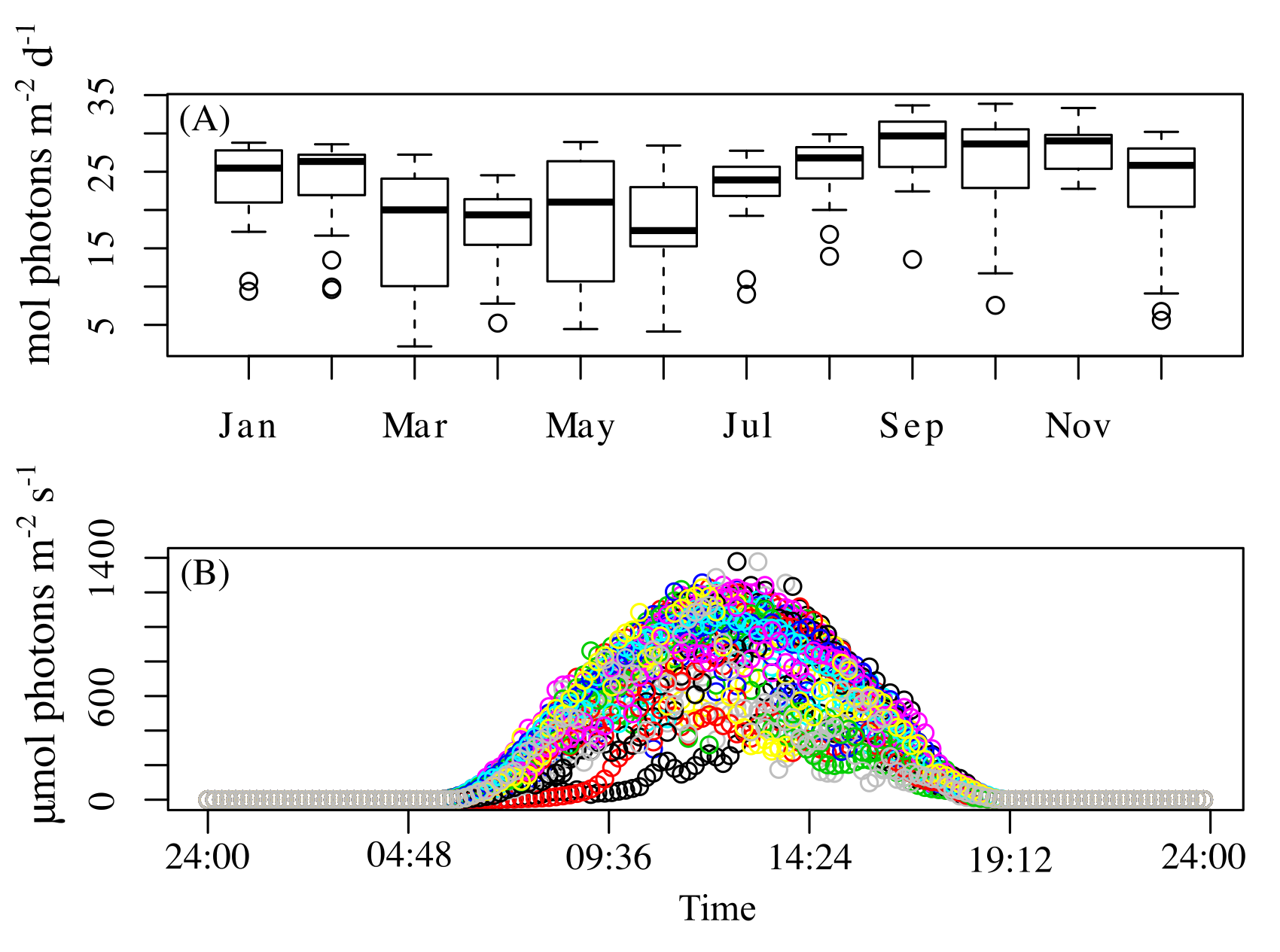

Supplement: S2 Fig — Davies Reef (A) mean daily light integrals (mol photons m-2 d-1) for May 2011 to May 2012 at 0.8m and (B) instantaneous PAR (μmol photons m-2 s-1) over the day in January 2012, colours representing the different days of the month. Data obtained via the AIMS Weather Station Program at: https://apps.aims.gov.au/metadata/view/076c8641-6e72-4be7-9eb7-e21145cc6525 and specifically http://data.aims.gov.au/aimsrtds/datatool.xhtml?from=1980-01-01&thru=2018-06-25&channels=9272,9273. (TIF) [file pone.0203882.s005.tif]
